# Earthworm-Driven Changes in Soil Chemico-Physical Properties, Soil Bacterial Microbiota, Tree/Tea Litter Decomposition, and Plant Growth in a Mesocosm Experiment with Two Plant Species

**DOI:** 10.3390/plants12061216

**Published:** 2023-03-07

**Authors:** Adriano Sofo, Mohammad Yaghoubi Khanghahi, Maddalena Curci, Francesco Reyes, Maria J. I. Briones, Judith M. Sarneel, Domenico Cardinale, Carmine Crecchio

**Affiliations:** 1 Department of European and Mediterranean Cultures: Architecture, Environment and Cultural Heritage (DiCEM), Università degli Studi della Basilicata, Via Lanera 20, 75100 Matera, Italy; 2 Department of Soil, Plant and Food Sciences, Università degli Studi di Bari Aldo Moro, Via Amendola 165/a, 70126 Bari, Italy; 3 Department of Life Sciences, Università degli Studi di Modena e Reggio Emilia, Via Giovanni Amendola 2, 42122 Reggio Emilia, Italy; 4 Department of Ecology and Animal Biology, Universidade de Vigo, 36310 Pontevedra, Spain; 5 Department of Ecology and Environmental Sciences, Linnaeus väg 6, Umeå Universitet, 90187 Umeå, Sweden; 6 Independent Researcher, 75100 Matera, Italy

**Keywords:** carbon/nitrogen ratio, *Eisenia* sp., olive litter, soil bacteria, soil chemico-physical properties, soil sustainable management, Tea Bag Index

## Abstract

Earthworms and soil microorganisms contribute to soil health, quality, and fertility, but their importance in agricultural soils is often underestimated. This study aims at examining whether and to what extent the presence of earthworms (*Eisenia* sp.) affected the (a) soil bacterial community composition, (b) litter decomposition, and (c) plant growth (*Brassica oleracea* L., broccoli; *Vicia faba* L., faba bean). We performed a mesocosm experiment in which plants were grown outdoors for four months with or without earthworms. Soil bacterial community structure was evaluated by a 16S rRNA-based metabarcoding approach. Litter decomposition rates were determined by using the tea bag index (TBI) and litter bags (olive residues). Earthworm numbers almost doubled throughout the experimental period. Independently of the plant species, earthworm presence had a significant impact on the structure of soil bacterial community, in terms of enhanced *α*- and *β*-diversity (especially that of Proteobacteria, Bacteroidota, Myxococcota, and Verrucomicrobia) and increased 16S rRNA gene abundance (+89% in broccoli and +223% in faba bean). Microbial decomposition (TBI) was enhanced in the treatments with earthworms, and showed a significantly higher decomposition rate constant (*k*_TBI_) and a lower stabilization factor (*S*_TBI_), whereas decomposition in the litter bags (*d*_litter_) increased by about 6% in broccoli and 5% in faba bean. Earthworms significantly enhanced root growth (in terms of total length and fresh weight) of both plant species. Our results show the strong influence of earthworms and crop identity in shaping soil chemico-physical properties, soil bacterial community, litter decomposition and plant growth. These findings could be used for developing nature-based solutions that ensure the long-term biological sustainability of soil agro- and natural ecosystems.

## 1. Introduction

Soil fauna play a key role in soil C storage capacity, nutrient cycling, and hydrology, that in turn affect soil quality [1,2,3,4]. However, the importance of soil organisms, including earthworms, in the delivery of ecosystem services is often overlooked, but it should be considered in future land management strategies, as healthy soil is an important resource to be protected [5].

The importance of earthworms in agriculture has been studied since Darwin [6]. In agricultural soils, earthworms contribute to soil health, quality, and fertility, by increasing soil water and nutrient contents, soil microporosity, and aeration [1,7,8,9,10,11]. This occurs because soil fauna, and in particular, earthworms, impact soil’s physical, chemical, and microbiological properties [9,10,11]. Medina-Sauza et al. [12] concluded that the positive effects of earthworms on soil fertility (in terms of nutrient availability, plant-growth promotion by signal molecules, soil water, and C content) are mainly mediated via interactions with the microbial communities. As an example of earthworms-microorganisms’ feedbacks, the mucus secretion in the earthworm guts and deposited in their biogenic casts enhance the metabolism of plant-growth promoting soil microorganisms and soil biocontrol microbial agents [13,14]. In addition, earthworms are associated with increased C and N soil contents and a higher diversity of niches for microorganisms because of bioturbation [4,15].

The issue of changes in soil microbial community composition is a controversial and much disputed subject within the field of soil management and land use. Several studies thus far have linked the vertical and horizontal spatial distribution of soil microbes with the species of cultivated plants through the assessment of the rhizodeposits and the status of soil organic matter and nutrients [16,17]. Earthworms are known to be ecosystem engineers, since they can influence soil microorganisms’ structure by ameliorating micro-habitat, enhancing the surface area of the organic compound, feeding, and transporting microbes [18]. Overall, such direct (modifying the quantity or quality of the substrate) or/and indirect (varying the soil environment conditions) factors were considered the main agents affecting soil microbial structure, function, and stability [19,20]. Furthermore, earthworms significantly increase soil microbial biomass and microbial respiration, being the main agents responsible for microbiological soil fertility [1,7,11].

It is known that plant growth and development are affected by soil biota [21]. At the same time, plants can influence soil biota, including earthworms, which in turn accelerates the decomposition of plant litter [22,23,24]. The study of microbial communities has increased dramatically thanks to the use of 16S rRNA metabarcoding, which allows a rapid screening of microbial diversity [4,25]. Standardized protocols to measure litter decomposition include the Tea Bag Index (TBI) method, which provides an easy way to measure the decay of plant material by using two standard types of tea bags (green and red tea) [26]. The rates of organic matter decomposition measured with the TBI method have been found to be significantly related to microhabitat conditions, microbial diversity and types of agricultural practices adopted [27,28,29,30], but the combined influence of earthworms, soil microbes, soil organic matter, and plants on early-stage tea decomposition has never been investigated before.

In this study, we designed a mesocosm experiment to test if and to what extent the presence of earthworms (*Eisenia* sp.) affected (a) soil bacterial community composition, (b) litter decomposition, and (c) plant growth (*Brassica oleracea*, broccoli; *Vicia faba*, faba bean). The epigeic earthworm *Eisenia* sp. was chosen for its proven role in soil quality improvement, as it is also present in compost, and for its plant growth-promoting effects [3,31,32]. The two plant species were selected based on their opposite root architecture and morphology (thin, deep, and dense taproot in broccoli; fibrous, shallow, and diffuse root system in faba bean) [33], which, interacting with the earthworms, could be able to differently modify soil chemical composition and support different groups of soil bacteria.

## 2. Results

### 2.1. Earthworm Abundance, Soil Abiotic Properties and Decomposition Rates, and Plant Growth

The scanned images acquired throughout the experiment allowed us to check the survival, abundance, and activity of earthworms, confirming that they did not suffer in the pots (Supplementary Figure S1). The number and total weight of earthworms doubled in the broccoli treatments with earthworms (*BR*_earth_) and faba bean with earthworms (*FB*_earth_) from the beginning to the end of the experiment, with the highest final values (107 specimens and 32.94 g total weight) observed in the *FB*_earth_ treatment (Table 1).

**Figure 1:**
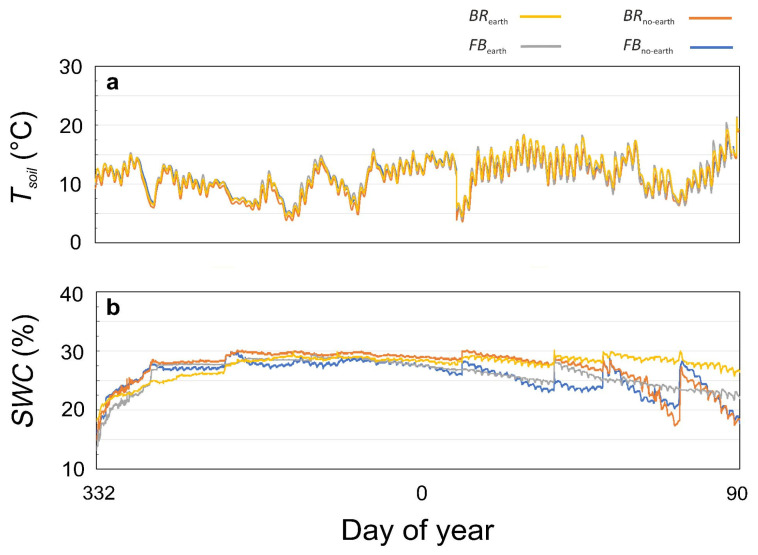
(**a**) Soil water content (*SWC*) and (**b**) soil temperature (*T*_soil_) measured throughout the experimental period in soils under different treatments, consisting of broccoli with earthworms (*BR*_earth_: yellow), broccoli without earthworms (*BR*_no-earth_: red), faba bean with earthworms (*FB*_earth_: grey), and faba bean without earthworms (*FB*_no-earth_: blue).

The values of soil temperature (*T*_soil_) did not significantly change among the treatments (Figure 1a). Within each treatment, a certain degree of variation in soil water content (*SWC*) was observed after a rainfall event, more pronounced in the second half of the trial, when plants were bigger, and in the treatments without earthworms (Figure 1b). The treatments with earthworms, especially *BR*_earth_, maintained more stable *SWC* values throughout the experiment and a higher mean *SWC* in the second half of the experiment compared to the treatments without earthworms (+7% in *BR*_earth_ and +5% in *FB*_earth_) (Figure 1b).

**Figure 2:**
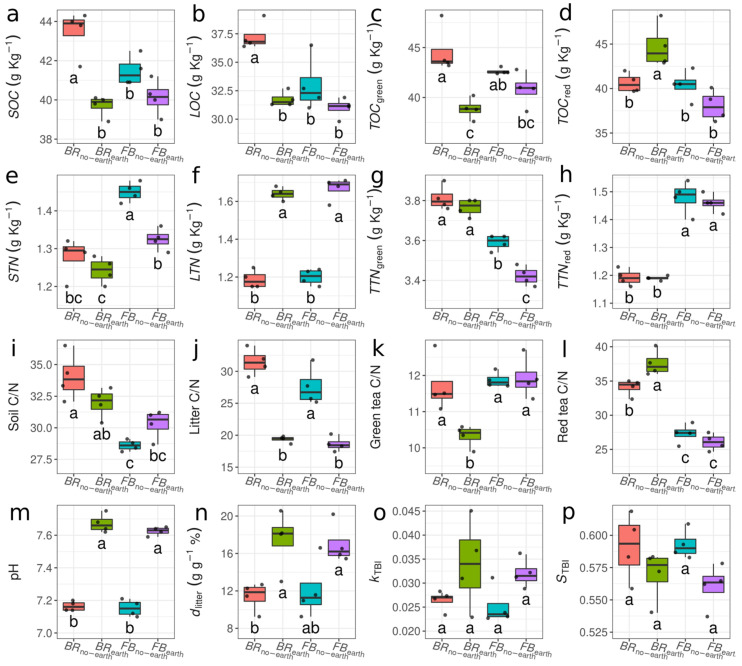
Boxplots of soil chemico-physical parameters in soil and litter under different treatments, consisting of broccoli with earthworms (*BR*_earth_), broccoli without earthworms (*BR*_no-earth_), faba bean with earthworms (*FB*_earth_), and faba bean without earthworms (*FB*_no-earth_). (**a**) Soil organic carbon (*SOC*); (**b**) litter organic carbon (*LOC*); (**c**) organic carbon in green tea bags (*TOC*_green_); (**d**) organic carbon in red tea bags (*TOC*_red_); (**e**) soil total nitrogen (*STN*); (**f**) litter total nitrogen (*LTN*); (**g**) total nitrogen in green tea bags (*TTN*_green_); (**h**) total nitrogen in red tea bags (*TTN*_red_); (**i**) soil carbon to nitrogen ratio (Soil C/N); (**j**) litter carbon to nitrogen ratio (Litter C/N); (**k**) carbon to nitrogen ratio in green tea bags (Green tea C/N); (**l**) carbon to nitrogen ratio in red tea bags (Red tea C/N); (**m**) soil pH (pH); (**n**) percentage of litter decomposed (*d*_litter_); (**o**) decomposition constant of tea bags (*k*_TBI_); (**p**) stabilization factor of tea bags (*S*_TBI_). Different letters indicate significant differences after two-way ANOVA followed by Tukey’s HSD test.

The presence of earthworms was generally associated with changes in the soil and litter chemico-physical parameters, although the differences between treatments were not always statistically significant (Figure 2, Supplementary Table S2). Overall, soil organic carbon (*SOC*), soil total nitrogen (*STN*), and C/N ratio decreased compared to the initial conditions (Supplementary Table S1). The presence of earthworms reduced the organic C content in the soil, litter bags, and green tea bags, significantly in both species for green tea bags, while only in broccoli for the other two variables (Figure 2a–c). Here the interaction terms were barely significant and with modest *F* statistics for all three variables, while the main effect was higher due to the presence of earthworms and only second the species effect. No significant reduction was present for red tea bags, which on the contrary was associated to an increase in carbon content for broccoli (Figure 2d). The total N in the soil and green tea bags decreased with earthworms (significantly only in *FB*_earth_) (Figure 2e–g), with barely significant and low *F* values interaction terms, while with more consistent F statistics related to highly significant main effects of the presence of earthworms. Conversely, total N in the olive litter significantly increased for both plant species (Figure 2f), and without species nor interaction effects, while the total N in the red tea bags remained stable (with the sole difference due to the species, Figure 2h). The C/N ratios mirrored the observed differences in C and N contents, with lower values in the presence of earthworms (Figure 2i–l), especially for *BR*_earth_, with the exceptions of soil C/N in faba bean (Figure 2i) and red tea C/N in broccoli (Figure 2l). More in depth, litter C/N was affected solely by earthworms; red tea C/N moderately by the interaction between species and presence of earthworms, but mainly by the plant species; while soil and green tea C/N largely by the interaction between the two (Supplementary Table S2).

Soil pH values were very similar in all treatments at the beginning of the experiment (on average 7.4) (Supplementary Table S1), but significantly increased by the end of the trial in the pots containing earthworms (7.67 in *BR*_earth_ and 7.63 in *FB*_earth_), in respect to pots without earthworms (7.17 in *BR*_no-earth_ and 7.15 in *FB*_no-earth_) (Figure 2m). The values of soil pH, *SOC*, *STN*, *TOC*_green_, *TTN*_green_, *TOC*_red_, *TTN*_green_, *LOC*, and *LTN* measured at the beginning for the experiment are shown in Supplementary Table S1.

Earthworms significantly increased both the percentage of litter decomposed (*d*_litter_) and tea decomposition constant (*k*_TBI_) (Figure 2n,o), but decreased tea stabilization factor (*S*_TBI_) (Figure 2p). More specifically, *d*_litter_ was enhanced in the treatments with earthworms by about 53% in *BR*_earth_ and 41% in *FB*_earth_, compared to the respective treatments without earthworms (Figure 2n). Interestingly, although not surprising, while differences in variables related to the C and N dynamics were generally due, to some extent, to an interaction between plant species and the presence/absence of earthworms, the other soil variables (pH, *d*_litter_, *k*_TBI_, and *S*_TBI_) were affected solely by the presence of the earthworms (no significant interaction nor species effects).

Significant differences in shoot growth due to earthworms were found only in faba bean (Table 2). Regarding root traits, root maximum length, and root fresh weight were influenced by the presence of earthworms both in broccoli and faba bean (Table 2).

### 2.2. 16S rRNA Metabarcoding Analysis

#### 2.2.1. α- and β-Diversity

**Figure 3:**
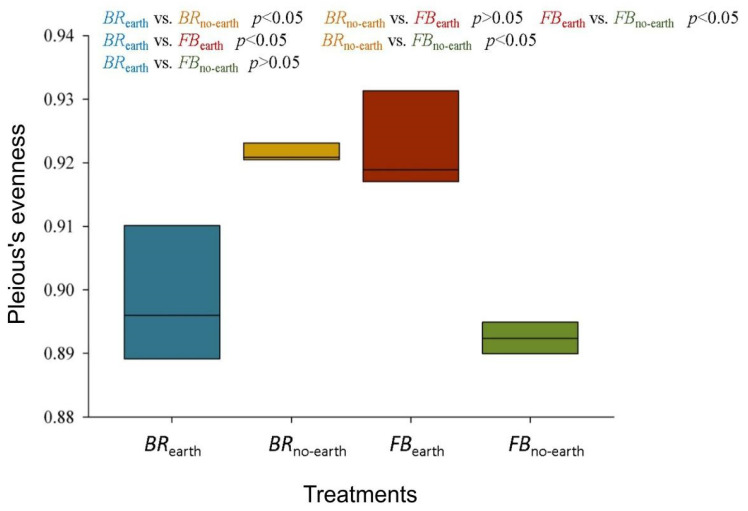
*α*-diversity index (evenness) for the bacterial community of soils under different treatments, consisting of broccoli with earthworms (*BR*_earth_), broccoli without earthworms (*BR*_no-earth_), faba bean with earthworms (*FB*_earth_), and faba bean without earthworms (*FB*_no-earth_). Statistical significance was determined using the Kruskal–Wallis test. The measures within each treatment are in triplicate.

The *α*-diversity analysis revealed a significant difference between treatments in terms of Pielou’s evenness index based on the Kruskal–Wallis test (Figure 3). In addition, the bacterial community in *BR*_earth_ were clearly distinct from those in *BR*_no-earth_ and *FB*_earth_ at *p* < 0.05 (Figure 3). Similarly, the Kruskal–Wallis test revealed a significant difference (*p*-value = 0.036) between *BR*_no-earth_ and *FB*_earth_, as well as between *FB*_no-earth_ and *FB*_earth_ (*p* < 0.05).

**Figure 4:**
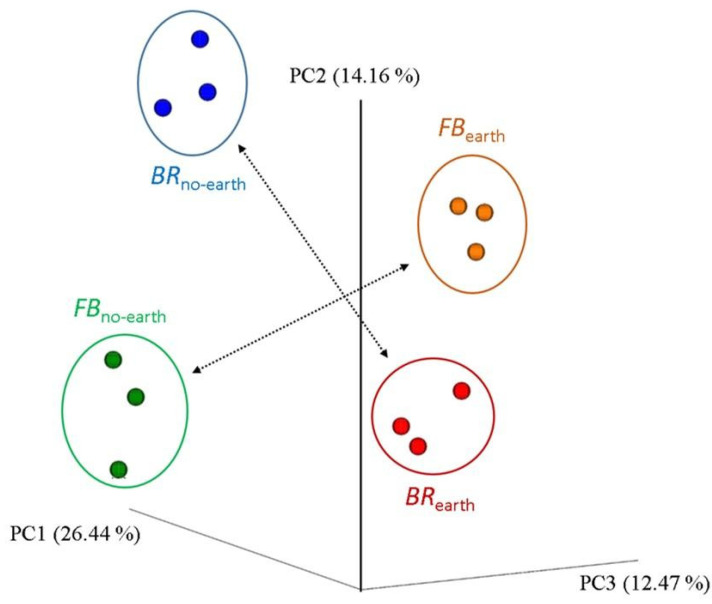
Principal coordinate analysis (PCoA) plots of *β*-diversity, calculated using the weighted UniFrac metric, for the bacterial community of soils under different treatments, consisting of broccoli with earthworms (*BR*_earth_), broccoli without earthworms (*BR*_no-earth_), faba bean with earthworms (*FB*_earth_), and faba bean without earthworms (*FB*_no-earth_). The proportion of the data variation are displayed as axis percentages. The measures within each treatment are in triplicate.

The extent of differences and similarities among the bacterial communities was also explored using *β*-diversity analysis. The principal coordinate analysis (PCoA) plot allows visualizing the existence of differences in *β*-diversity between the bacterial communities in the different treatments based on Weighted UniFrac distance (phylogenetic method) (Figure 4). 

**Figure 5:**
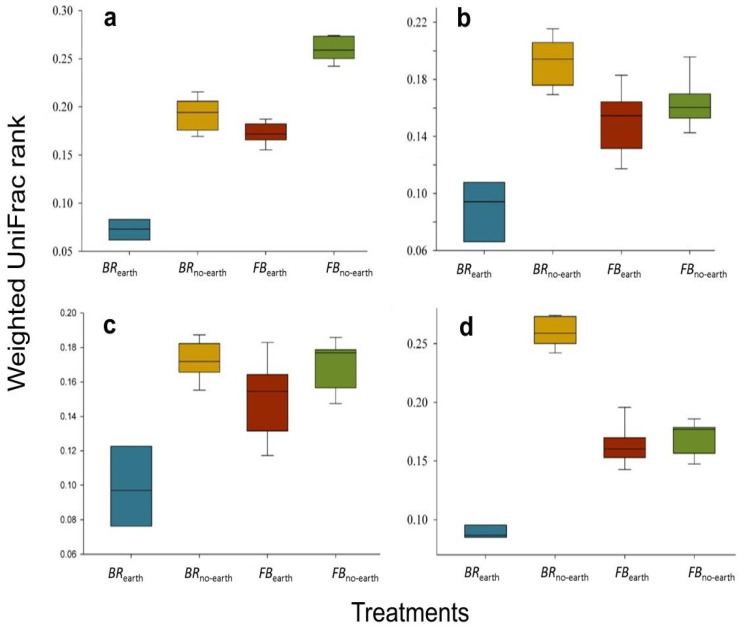
Distance of the group of treatments in (**a**) broccoli with earthworms (*BR*_earth_), (**b**) broccoli without earthworms (*BR*_no-earth_), (**c**) faba bean with earthworms (*FB*_earth_), and (**d**) faba bean without earthworms (*FB*_no-earth_) based on the analysis of similarities (ANOSIM). The estimated *R* value (=0.99) proved dissimilarity between groups (*p* < 0.001). The measures within each treatment are in triplicate.

Significance was tested using ANOSIM with 999 permutations (*p*-value = 0.001). In addition, the treatments differed significantly, based on the analysis of similarities (*R* value = 0.99, *p* < 0.001) through weighted UniFrac distance (Figure 5). 

#### 2.2.2. Bacterial Community Composition

As shown in Supplementary Table S3, a total of 11 bacterial phyla (with a relative abundance > 1%) were identified in soil samples. Almost all of these phyla were significantly affected by the simple and/or interaction effects of the presence of earthworms and the type of cultivated plants’ treatments, based on the two-way ANOVA analysis. The analysis of Tukey’s HSD test (Table 3) illustrated that Proteobacteria, Bacteroidota, Chloroflexi, Myxococcota, and Verrucomicrobia had a higher relative abundance in the *BR*_earth_ than in other sample types, while Patescibacteria, Firmicutes, Acidobacteria, and Gemmatimonadetes in *FB*_no-earth_, and Actinobacteriota in *BR*_no-earth_ had a higher proportion. In this regard, the presence of earthworms significantly increased the abundance of Proteobacteria, Bacteroidota, Myxococcota, and Verrucomicrobia in the soil samples (+15.3, +15.6, +66.9, and +94.6%, respectively, in *BR*_earth_; +14.8, +13.4, +31.9, and +6.6%, respectively, in *FB*_earth_), compared to their abundances in the treatments without earthworms (Table 3). In contrast, the abundances of Actinobacteriota, Firmicutes, Patescibacteria, Acidobacteria, and Gemmatimonadetes significantly decreased in *BR*_earth_ (–61.2, –102.1, –14.8, –23.4, and –67.4%, respectively) and *FB*_earth_ (–12.8, –48.7, –62.4, –24.5, and –20.6%, respectively), compared to the values in the treatments without earthworms (Table 3). 

Furthermore, the abundances of 11 bacterial families out of 12 detected families (with a relative abundance > 2%) were significantly changed by the interaction effects of the treatments (Table S3). Accordingly, some of them became relatively more abundant in soils with earthworm activity when compared to those without earthworms, such as BIrii41 (+94.6 and +135.7%), Devosiaceae (+59.4 and +25.4%), Pirellulaceae (+16.9 and +20.8%), Microscillaceae (+41.7 and +18.4%), and Microbacteriaceae (+80.7 and +135.1%) in broccoli and faba bean, respectively (Table 3; Supplementary Figure S3). On the other hand, the changed abundance of some bacterial families under earthworm activity led to a significant reduction in the abundance of Bacillaceae, Streptomycetaceae, Saprospiraceae, and Streptosporangiacea, which were 1.7, 8.4, 3.5, and 8.2 times lower in *BR*_earth_ compared to *BR_no-_*_earth_, and 1.1, 0.9, 3.4, and 2.2 times lower in *FB*_earth_ compared to *FB_no-_*_earth_, respectively (Table 3 and Supplementary Figure S3). Moreover, Planctomycetota phylum and Microscillaceae family were significantly influenced neither by simple effects nor by the interaction effects of the treatments (Table 3 and Supplementary Table S3). 

At the genus level, the most representative effect of earthworm activity was an increase in the abundance of many genera, the most important of which were *BIrii41* (+95.6 and +136.9%), *Devosia* (+65.7 and +59.6%), *Flavobacterium* (+431.1 and +961.5%), and *Ohtaekwangia* (+330.6 and 1194.2%) in *BR*_earth_ and *FB*_earth_, respectively (Supplementary Figure S4). Conversely, earthworm activity reduced the abundance of some other genera, such as *Bacillus*, *Nonomuraea*, and *Streptomyces*, by 170.7, 947.4, and 740.9% in *BR*_earth_, and 102.8, 112.9, and 88.9% in *FB*_earth_, respectively (Supplementary Figure S4). 

According to Ward’s clustering method, the bacterial communities at the family and genus levels (relative abundance > 1%) clustered into several groups in which the *BR*_earth_ treatment was clearly separated from the rest, while *BR*_no-earth_ and *FB*_no-earth_ treatments were clustered into one group (Supplementary Figures S3 and S4). On the basis of the ANCOM method, of the 329 genera identified in the broccoli treatments, only *Opitutus* (W = 202), *Rubritalea* (W = 165), *Terrimonas* (W = 164), uncultured-*Verrucomicrobiaceae* (W = 156), and Bly10 (W = 154) were present in *BR*_earth_ (Supplementary Figure S5), whereas of the 285 genera found in faba bean treatments, *Paracoccus* (W = 160), *Kazania* (W = 134), *Caenimonas* (W = 121), and S-70 (W = 118) were present only in *FB*_earth_ (Supplementary Figure S6).

#### 2.2.3. Quantitative PCR of the 16S rRNA Gene

The results of two-way ANOVA analysis revealed a significant change in 16S rRNA gene copy numbers in soil in response to the earthworm activity (*p* < 0.01) and the interaction effects of treatments (*p* < 0.05) (Supplementary Table S3). In this regard, the larger set of significant 16S rRNA gene copy numbers in soil samples were recorded in *BR*_earth_ (1.15 × 10^10^ copies g^−1^ of soil) and *FB*_earth_ (1.26 × 10^10^ copies g^−1^ of soil), which were significantly higher than *BR*_no-earth_ (+89%) and *FB*_no-earth_ (+223%), respectively. However, the type of cultivated plant had no significant effect on the ribosomal gene abundance in the examined soils (Supplementary Figure S7 and Table S3).

### 2.3. Multivariate Analysis

Canonical Correspondence Analysis (CCA) showing the relationships between the bacterial community structure at phylum level (relative abundance > 1%) and soil and litter parameters is reported in Supplementary Figure S8. Most of the total variance was accounted for by the first two components (88%). The results showed that the different treatments grouped into four different clusters: *BR*_earth_, *FB*_earth_, and *FB*_no-earth_ along the first principal component axis, while *BR*_no-earth_ along the second principal component axis. The *BR*_earth_ cluster had strongest relationships with soil pH, *LTN*, *d*_litter_, Myxococcota, and Proteobacteria. The *BR*_no-earth_ cluster showed a close relationship with *LOC*, *SOC*, *TOC*_green_, litter C/N, Firmicutes, and Actinobacteriota. The *FB*_no-earth_ cluster was associated with *STN*, *TTN*_red_, and Patescibacteria, while *FB*_earth_ cluster does not appear to be influenced by any chemical parameter.

## 3. Discussion

### 3.1. Earthworm Presence and Plant Species Affected Soil Bacterial Community via Different Soil Ecological Niches

Our results indicate that the observed changes in soil bacterial community were associated with plant responses to earthworm activities (Table 3; Figure 3, Figure 4 and Figure 5). The outcome of the analyses of Bacteria *α*- and *β*-diversity for each plant species showed that bacterial community structure shifts were more pronounced for broccoli than for faba bean, especially in the presence of earthworms (Figure 3 and Figure 4). Yaghoubi Khanghahi et al. [34] found that a different plant phenotypic response provides an altered habitat, probably via adjusted root architecture. In our study, these effects were more pronounced in soils with broccoli, with a thin, deep, and dense taproot, compared to faba bean, which has shallow, diffuse, and longer roots [4]. The different microbial changes between the two plant species (Figure 3, Figure 4 and Figure 5) could also be due to different *SWC* values (higher in broccoli due to the deeper root penetration into the soil) (Figure 1) and soil C/N (lower in faba bean due to N fixation) (Figure 2i). Recent studies by Gong et al. [35] and Zhang et al. [36] also support the view that soil microbial communities could be regulated by soil chemico-physical properties, including nutrient factors (e.g., C/N ratio, total N, total P, etc.), and non-nutrient factors (e.g., vegetation cover, soil aggregate stability, pH, etc.). In addition to the direct impacts of earthworms on soil bacterial community and diversity [37], they can enhance soil bacterial activities measured as microbial respiration [38] and increase soil microbial biomass and enzyme activity [36].

The findings observed here (Table 1; Supplementary Figures S5 and S6) mirror those of previous studies that reported an increasing abundance of some copiotrophic bacterial groups in response to the earthworm activities [12,35], such as Proteobacteria and Bacteroidota (with the dominant families of Flavobacteriaceae, Devosiaceae, Microscillaceae, and R7C24) (Table 1; Supplementary Figures S5 and S6). In accordance with the hypothesis introduced by Männistö et al. [39], the increase in the ratio between Proteobacteria and Acidobacteria in the earthworm-containing soils, compared to the treatments without earthworms (+42% in *BR*_earth_ and +43% in *FB*_earth_), indicates better copiotrophic conditions in the former treatments, and confirms the ability of earthworms to alter the structure of the soil bacterial community under the two plant species studied here. Therefore, reductions in soil nutrient content and lower quality and quantity of organic C in those soils with earthworms led to the redundancy of ribosomal RNA gene copy numbers, as indicated by Männistö et al. [39] and Khanghahi et al. [40].

Conversely, the treatments lacking earthworms promote more oligotrophic Bacteria phyla such as Firmicutes, Patescibacteria, Acidobacteria, and Gemmatimonadetes (Table 1; Supplementary Figures S5 and S6), and also a lower number of 16S rRNA gene copies (Supplementary Figure S7). Moreover, the increase in Actinobacteria in the treatments without earthworms could be related to higher amounts of soil organic C and total N [41]. This could explain their role as important decomposers of complex organic compounds with a great capability to degrade recalcitrant organic matter [40]. In relation to this, He et al. [42] reported a significant reduction in the biomass of soil Actinobacteria in the presence of earthworms. A possible explanation for this result may be the selective feeding and digestion of some specific taxa by earthworms, which led to reductions in the abundance of some bacterial groups [42,43].

In addition, plant–microbe interactions [44] and microbe–microbe symbiotic relationships occurring in the rhizosphere [34] should also be taken into account together with their direct impact on soil bacterial community. This was evident in the case of Flavobacteriaceae, the relatively most abundant family in our experiment, whose abundance changed in response to earthworm activity under broccoli and faba bean cultivation (Table 1; Supplementary Figures S5 and S6). In particular, the abundance of this bacterial family around the rhizoplane has been reported to be higher than in the rhizosphere and bulk soil and seems to be closely related to the type of cultivated plants [45].

### 3.2. Earthworm-Driven Changes in Soil Chemico-Physical Parameters Resulted in Different Litter Decomposition Rates and Plant Growth

In our study, earthworms were responsible for most of the chemico-physical changes observed in the soil (Figure 1 and Figure 2). The earthworm influence on soil macroporosity [5,8] was likely the main factor responsible for the observed higher *SWC* content and stability (particularly evident during the second half of the experimental period) observed in both *BR*_earth_ and *FB*_earth_, compared to the respective values found in *BR*_no-earth_ and *FB*_no-earth_ (Figure 1b). In the case of soil pH (Figure 2m), it is known that earthworm casts (small heaps of egested materials deposited on the soil surface and earthworm burrows) and mucus secretions can increase the soil pH [32,46], even if this action was likely attenuated by the acidifying actions of root exudates. 

The excrements of earthworms are also rich in nutrients and microorganisms [1,7]. Therefore, they can be considered a natural fertilizer that contains five times more N, seven times more phosphorus, and eleven times more calcium than the surrounding soil [13,14]. The observed values of *STN* were higher in *BR*_no-earth_ and *FB*_no-earth_, compared to the respective values measured in *BR*_earth_ and *FB*_earth_ (Figure 2e), indicating that most of the readily available soil N was absorbed by roots and/or used for microbial growth. Interestingly, *STN* was significantly higher in the faba bean treatments (*FB*_earth_ and *FB*_no-earth_) compared to the broccoli ones (*BR*_earth_ and *BR*_no-earth_) (Figure 2e), possibly as a result of the N-fixing activity of root nodules in the legume. This also attenuated the differences in the C/N ratio in the faba bean soils (Figure 2i).

The earthworm-induced changes in soil chemico-physical parameters were related to tea and olive litter decomposition rates (Figure 2n–p). As hypothesized, earthworms accelerated the decay processes as confirmed by the increments in the decomposition rate constant (*k*_TBI_), a decreased stabilization factor (*S*_TBI_), and greater litter mass losses (*d*_litter_) in the earthworm treatments (Figure 2n–p). That earthworms enhance the decomposition of different types of organic matter aligns well with the observed correlation between earthworm density and (tea) decomposition in the field [18,28]. In a greenhouse study, however, no direct effect of earthworms on decomposition rates and stabilization was found [47]. This may indicate that species identity and ecological grouping (anecic *Lumbricus terrestris* in the previous study vs. epigeic *Eisenia* sp. in our study) or earthworm density might have also played a role (0.25 g L^−1^ vs. 0.5 g L^−1^ in our study) (Table 1). 

The changes in soil chemico-physical parameters and decomposition rates (Figure 1 and Figure 2) and the different structure of soil bacterial community due to earthworm presence (Table 3; Figure 3, Figure 4 and Figure 5) determined an accelerated plant growth, particularly evident in the root system (Table 2).

### 3.3. Relationships between Soil and Litter Properties and Bacterial Community Composition

The CCA (Supplementary Figure S8) demonstrated that *BR*_no-earth_ is more influenced by organic carbon (e.g., *LOC*, *SOC*, litter C/N, and *TOC*_green_), while *FB*_no-earth_ is more related to nitrogen (e.g., *STN*, *TTN*_red_). Thus, in the treatments without earthworms, the plant species had a predominant effect. In the same CCA graph, *FB*_earth_ is located in the middle and seems to be the least affected by the chemical aspects, as only *k*_TBI_ and *S*_TBI_ were close (opposite to each other, indicating an inverse correlation).

Regarding the bacterial component parameters, they were all fairly close to each other but it is possible to identify some phyla that are more correlated with some treatments. Specifically, Bacteriodota, Myxococcoya, Proteobacteria, and Verrucomicrobiota were influenced by earthworms (evident in *BR*_earth_ but also in *FB*_earth_) much more than by the type of plant species. Planctomycetota, Actinobacteriota, and Firmicutes were affected by *BR*_no-earth_, while Acidobacteriota and Patescibacteria by *FB*_no-earth_. In particular, Patescibacteria were related with total nitrogen and they also were one of the phyla that differentiate soil samples with and without earthworms in ANCOM analysis (Supplementary Figures S5 and S6).

## 4. Materials and Methods

### 4.1. Experimental Set-Up

The experimental area (Trani, BT, Puglia Region, Italy; 41°16′25″32 N, 16°24′58″32 E) is characterized by a semi-arid climate, with an average annual rainfall of 595 mm (1995–2021) and a mean annual temperature of 16.0 °C. The trial was carried out outdoors in the Autumn–Spring 2020–2021 (November–March). The experiment was performed on potted plants, using the same soil type and under identical climatic conditions in a rainfed regime. This allowed the elimination of any indirect effects due to initial leaf litter composition, soil type, or climate regime.

On 28 November 2020, three-week-old seedlings of broccoli (*Brassica oleracea* L.) and faba bean (*Vicia faba* L.), which were germinated in peat under controlled conditions (20 °C and 16:8 h light:dark photoperiod), were planted in 30 L conical pots filled with local topsoil coming from an adjacent olive orchard (depth = 0–30 cm; sandy clay texture, with 45% sand, 14% silt, and 41% clay) that was chemico-physically characterized (electric conductivity = 0.159 mS cm^−1^; total CaCO_3_ = 4.2%, active CaCO_3_ = 1.0%; assimilable phosphorus = 5 mg kg^−1^; cation exchange capacity = 11.70 meq 100 g^−1^, base saturation = 100%, Mg/K ratio = 1.78) (C/N and pH data in Supplementary Table S1). Pots were incubated outside and exposed to the elements. Soil water content (*SWC*) was not statistically different between pots at the beginning of the experiment (Figure 1b). Eight pots per each plant species were established, each one with one plant seedling. Half of the pots (four of each plant species) received approximately 15 g (fresh weight) of mature, clitellated earthworms (*Eisenia* sp.) that were previously counted, weighed, and then gently mixed to the soil (earthworm treatments) (Table 1); the remaining four pots for each plant species did not contain earthworms (control treatments). Earthworms were purchased from a local supplier (Fattoria Gallorosso Ssa; Matera, Italy). This resulted in four treatments replicated four times (broccoli with earthworms, *BR*_earth_; faba bean with earthworms, *FB*_earth_; broccoli without earthworms, *BR*_no-earth_, and faba bean without earthworms, *FB*_no-earth_). The experiment ended on 31 March 2021, giving a total time of 121 days.

### 4.2. Earthworm Abundance and Imaging

In *BR*_earth_ and *FB*_earth_, earthworms were counted and weighed at the start (Supplementary Figure S1) and at the end of the experiment (31 March). After emptying the pots on a plastic sheet, the earthworms were washed with tap water to remove any soil particles.

In two replicates of each *BR*_earth_ and *FB*_earth_, an image scanner (Canon CanoScan D646U; Canon Electronics Inc., Tokyo, Japan) without the upper cover lid, was placed in a sealed plastic bag and diagonally placed in the partially filled pots, with the connecting USB cable emerging from it (Supplementary Figure S2a,b). Thereafter, the pots were completely filled with soil. This allowed the imaging of the soil inside the pot and of earthworm activity. Pictures were taken on six days (1 December, 3 January, 1 February, 28 February, 15 March, and 30 March) (Supplementary Figure S1).

### 4.3. Soil Bacterial Community Structure

At the end of the trial (31 March), plants and soil were removed from the pots and placed on sterile plastic sheets. The soil was manually mixed using sterile gloves to make up a composite soil sample of about 1 kg. This sampling technique increased soil homogeneity and the resulting soil samples included the soil around the mesh/litter bags, the soil surrounding the roots, and the bulk soil where earthworms were predominant. After removing the visible root residues, the soil composite samples were stored in sterile plastic bags at 4 °C and used within three days. Aliquots of the sampled soils were stored and then dried for the following chemical analyses.

Soil DNA was extracted from 0.5 g of soil by using the soil DNA extraction kit (MP Biomedicals^TM^ FastDNA^TM^ Spin Kit). The DNA quality and concentration were checked using the NanoDrop spectrophotometer (ND-1000, EuroClone, Italy). All samples were diluted to a concentration of 20 ng mL^−1^ and stored at −20 °C until the sequencing procedure. PCR of V3-V4 hypervariable regions of the 16S rRNA was performed by universal primers: 341F (5′-CCTACGGGNBGCASCAG-3′) and 805R (5′-GACTACNVGGGTATCTAATCC-3′). PCR and sequencing procedure was carried out by IGA Technology service (Udine, Italy, https://igatechnology.com/ (accessed on 13 September 2022) using an Illumina MiSeq next-generation sequencer (Illumina, San Diego, CA, USA) with 300 bp paired-end mode.

qPCR analysis was carried out to estimate bacterial rRNA gene copy numbers with the 515F/806R primer pairs. qPCR amplification in 20 μL volume contained 10 μL of iTaq Universal SYBR Green Supermix (2X; Bio-Rad, Hercules CA, USA), 0.4 μL of each primer (10 μM), 0.6 μL of BSA, 2 μL of template DNA, and 6.6 μL of nuclease-free water. The cycling conditions for the qPCR assay entailed enzyme activation at 95 °C for 3 min, followed by 40 cycles of denaturation at 95 °C for 15 s, annealing at 57 °C for 60 s, and extension at 72 °C for 12 s. Amplification specificity was assessed by melting curves which were followed by ramping the temperature from 60 to 95 °C, with a reading every 0.5 °C. Standard curves were obtained using a series of 10-fold dilutions of PCR products amplified from the positive control samples which were extracted from the agarose gels using the NucleoSpin^®^ Gel and PCR Clean-up (MACHEREY-NAGEL GmbH & Co. KG, Düren, Germany) and quantified by Nanodrop spectrophotometer (ND-1000, EuroClone, Milan, Italy).

### 4.4. Litter Decomposition Rates

#### 4.4.1. Tea Bag Index

On 28 November 2020, one green tea bag (*Camelia sinensis*; n. EAN 87 10908 90359 5; Lipton) and one red tea (rooibos) bag (*Aspalanthus linearis*; n. EAN 87 22700 18843 8; Lipton Unilever, Glasgow, UK) were weighed and inserted 10 cm apart at 10 cm soil depth in each pot. The tea bags were retrieved after 90 days on 27 February 2021. Soil particles and roots were removed, and the tea bags were oven-dried at 70 °C for 48 h and weighed. The Tea Bag Index (TBI) was calculated using the mass losses of red and green tea [21]. The TBI describes the decay of labile material fractions expressed by the decomposition rate constant (*k*_TBI_) indicates the rate at which labile material fractions are decomposed and the stabilization factor (*S*_TBI_) that is a proxy for how much the labile fraction is not decomposed in the early stages of the decomposition process. Using the mass loss of the tea bags, we calculated the Tea Bag Index (TBI) using a two-phased decomposition model:

(1) $$M\left(t\right)=ae^{-kt}+\left(1-a\right)$$

where *M*(t) is the mass proportion of the substrate after incubation time *t* in days, *a* is the decomposed labile fraction of the litter, *1 − a* is the remaining fraction, and *k* is the decomposition rate of the labile material fraction. After three months, green tea will lose very little mass with longer incubation and the remaining mass thus allows the decomposed fraction of green tea (*a_g_*) to be calculated:

(2) $$a_{g}=1-\frac{M\left(t\right)}{M_{g}\left(0\right)}$$

where *M_g_*(0) is the starting mass of green tea. 

The fraction of the labile material that is not decomposed by microorganisms, but stabilized (*S*_TBI_), was then calculated using the hydrolysable fraction of green tea (*H_g_*):

(3) $$S_{\mathrm{TBI}}=1-\frac{a_{g}}{H_{g}}$$

Assuming that *S*_TBI_ is equal for red and green tea, and using the hydrolysable fraction of red tea (*H_r_*), the decomposed fraction of red tea (*a_r_*) was calculated, from which *k*_TBI_ was derived using Equation (1).

(4) $$a_{r}=H_{r}\;\left(1-S_{\mathrm{TBI}}\right)$$

#### 4.4.2. Litter Bags

Litter bags with a size of 20 × 15 cm were prepared using non-decomposable tulle fabric (TFT Spa; Segrate, Milan, Italy) and filled with chemically characterized leaf litter (organic carbon = 42.10 g kg^−1^; total nitrogen = 5.75 g kg^−1^) collected from an adjacent olive orchard, dried at 25 °C for 15 days and weighed separately. The bag mesh size of 1 mm allowed microorganisms and small mesofauna to enter the bags but excluded macrofauna [2]. On 28 November 2020, the litter bags were buried at 10 cm depth in each pot and retrieved on 31 March 2021. Thereafter, the surrounding roots were cut off and the attached soil was carefully removed, and finally, the litter bags were oven-dried at 70 °C for 48 h and weighed. The difference between the initial and post-incubation total weights of the litter bags were used for calculating the percentage mass loss due to litter decomposition (*d*_litter_).

### 4.5. Chemico-Physical Analyses

Soil temperature (*T*_soil_) and *SWC* were monitored throughout the experiment at 10 cm depth and 10 min intervals in one replicate per treatment. Soil temperature was measured by DS18B20 digital sensors (Maxim Integrated, San Jose, CA, USA) calibrated by analogical thermometers (Brannan, Cleator Moore, UK). Soil moisture was measured by capacitive sensors (Seeed Studio, Shenzhen, China) (Supplementary Figure S2c,e) and expressed as a percentage of soil dry weight (drying at 105 °C for 24 h). All sensors were controlled by a board Arduino UNO with an integrated microcontroller ATMEGA328P (Arduino s.r.l., Monza, Italia), and data were recorded by a DS3231 datalogger (Adafruit Industries, New York, NY, USA) (Supplementary Figure S2c,d). 

As explained above, composite soil samples were taken from each pot at the end of the experiment (31 March). The soil was dried at 105 °C for 24 h, placed in a desiccator until a constant weight was reached, and then sieved through a 2 mm stainless steel sieve. The organic carbon and total nitrogen content in the soil (*SOC* and *STN*, respectively), tea bags (*TOC* and *TTN*, respectively), and litter (*LOC* and *LTN*, respectively) were determined at the end of the experiment (27 February 2021 only for *TOC* and *TTN*, whereas 31 March for the other parameters). Organic carbon was determined by the Walkley and Black method by oxidation at 170 °C with potassium dichromate (K_2_Cr_2_O_7_) in the presence of sulfuric acid (H_2_SO_4_), and the excess K_2_Cr_2_O_7_ was measured by Möhr salt titration, while total N was measured by the Kjeldahl method [48]. Soil pH was measured by a glass electrode (Basic 20^®^; Crison Instruments SA, Barcelona, Spain) by suspending soil in distilled water (1:2.5 soil to liquid phase ratio). For all these parameters, C/N ratios were calculated by dividing the values of organic carbon by total N. The values of *SOC*, *STN*, soil pH, *TOC*, *TTN*, *LOC*, and *LTN* were also measured at the beginning of the experiment (28 November) on random soil, tea bag, and litter samples.

### 4.6. Plant Growth Parameters

At the end of the experiment, plants were carefully removed from the pots after wetting the soil to avoid root damage. Shoots were separated from the roots by a scalpel. Shoot maximum height was measured using a ruler and then whole shoots (including stem, leaves, and fruits) were weighted (fresh weight). Then, the roots were cleaned by washing off the excess soil using tap water and slightly dried with an absorbent cloth. The root maximum length, which is an estimate of the rooting depth, was measured using a ruler and then the root fresh weight was recorded.

### 4.7. Bioinformatics 

For the 16S rRNA metabarcoding analysis, paired-end Illumina sequencing raw reads were imported into Microbial Ecology 2 software (QIIME2 2021.8 distribution, https://qiime2.org/ (accessed on 20 September 2022)) [49].

The total number of reads obtained from the sequencing run was 1,872,622. Forward and reverse reads (approximately 936,000 each) were quality filtered, denoised, paired-end reads merged using DADA2 pipeline [50] which includes the removal of chimeric reads. Further analyses were performed by Qiime2 at a sampling depth of 19,810 sequences per sample in order to normalize all samples to the size of the less abundant one, maintaining the richness of the dataset. A phylogenetic tree was generated by a phylogeny pipeline using the script “qiime diversity analysis align-to-tree-mafft-fasttree”. To compare the bacterial community structure of treatments, alpha diversity analyses were carried out on ASVs (amplicons sequence variants) data. We used the script “qiime diversity group significance” to test alpha diversity metrics (observed ASVs, Faith’s Phylogenetic Diversity, Shannon diversity index, and Pielou’s evenness index) and to compare both species richness and evenness within samples. Beta diversity was analyzed by “beta-group-significance” scripts by Weighted UniFrac distance matrix, which incorporates phylogenetic distances between observed organisms, to compare diversity in the community composition between treatments. A Naïve Bayesian classifier was used for taxonomic classification against the SILVA database (https://www.arb-silva.de/ (accessed on 12 October 2022)) using the script “qiime feature-classifier classify-sklearn”. Data analysis bar charts (Supplementary Figure S4) were created at phylum- and class-level taxonomic assignments for each replicate sample.

### 4.8. Statistical Analyses and Data Visualization 

The impact of the earthworms, in interaction with the plant species, on the soil and litter chemico-physical properties was assessed by means of two-way ANOVA followed by post hoc Tukey’s Honest Significant Difference (HSD) tests, in the R statistical environment (https://www.r-project.org/ (accessed on 31 October 2022)) to determine the effect of earthworm presence and plant species on the studied parameters. 

The *α*-diversity statistical analysis was conducted in Qiime2 using the Kruskal–Wallis test (Figure 3). The *β*-diversity statistical analysis was conducted in Qiime2 using the ANOSIM test with 999 permutations and visualized by PCoA plot (Figure 4). Ward’s clustering method, expressed by Euclidean distance, was used to compute the distance among bacterial community compositions in response to the presence of earthworms and species of cultivated plant (Figure 5). ANCOM (Analysis of Composition of Microbiomes) in Qiime2 [51] was carried out at the genus level using q2-composition pipeline after removing the zeros by q2-pseudocount. The volcano plots (Supplementary Figures S5 and S6) represent the ANCOM visualization where the *x*-axis is centered by log-ratio (clr) of F statistic and the W value of *y*-axis represents the number of rejected null hypotheses (that is, the average abundance of a given species in a group is the same as that in the other group).

Canonical Correspondence Analysis (CCA) was performed using the Past 4.12 software (https://past.en.lo4d.com/windows (accessed on 28 February 2023)) to relate the bacterial community composition to soil and litter characteristics.

## 5. Conclusions

Our findings allow us to conclude that earthworms had a significant impact on soil chemico-physical properties and soil bacterial community structure, and promoted litter decomposition and plant growth. The presence of earthworms allowed roots of both broccoli and faba bean to grow better in soils that accumulated high levels of nutrients. Even if this study has been conducted in mesocosms in order to have controlled conditions, at a larger scale and from an agricultural point of view, earthworms can be seen as a potential nature-based solution to ensure the sustainable use and conservation of soils, including adaptation and resilience to climate change, and for the long-term biological sustainability of soil systems. In the future, a repetition of this experiment with agricultural earthworm species (e.g., *L. terrestris*, *A. caliginosa*, *A. rosea*), also including perennial crops, would greatly enhance the potential of this study for transferring the results to practitioners, farmers, SMEs, policy makers and related end users for designing sustainable land use systems in different soils and climates. 

## Figures and Tables

**Table 1:** The number and total weight of earthworms were measured at the beginning and at the end of the trial in soils under different treatments, consisting of broccoli with earthworms (*BR*_earth_) and faba bean with earthworms (*FB*_earth_). Each value represents the mean (±SD) from four measurements (*n* = 4). Means followed by different letters within columns are significantly different at a 5% probability level (Tukey’s HSD test).

Date	Treatment	Earthworms (Number)	Earthworms (g)
28 November	*BR*_earth_	61 ± 6 b	14.56 ± 2.04 b
	*FB*_earth_	58 ± 2 b	14.47 ± 1.04 b
31 March	*BR*_earth_	101 ± 9 a	31.24 ± 4.49 a
	*FB*_earth_	107 ± 17 a	32.94 ± 3.99 a

**Table 2:** Maximum shoot height, shoot fresh weight, maximum root length, and root fresh weight in plants under different treatments, consisting of broccoli with earthworms (*BR*_earth_), broccoli without earthworms (*BR*_no-earth_), faba bean with earthworms (*FB*_earth_), and faba bean without earthworms (*FB*_no-earth_). Each value represents the mean (±SD) from four measurements (*n* = 4). Means followed by different letters within columns are significantly different at 5% probability level (Tukey’s HSD test).

Treatment	Maximum Shoot Height (cm)	Shoot Fresh Weight (g)	Maximum Root Height (cm)	Root Fresh Weight (g FW)
*BR*_earth_	16.9 ± 1.9 c	355.08 ± 76.89 a	69.0 ± 3.9 a	317.06 ± 29.13 a
*FB*_earth_	63.4 ± 5.4 a	393.12 ± 44.45 a	37.2 ± 3.2 c	192.00 ± 15.50 c
*BR*_no-earth_	15.3 ± 0.7 c	309.14 ± 45.40 ab	55.2 ± 3.8 b	270.08 ± 26.84 b
*FB*_no-earth_	51.0 ± 5.1 b	292.24 ± 34.84 b	31.2 ± 2.1 d	157.78 ± 23.21 d

**Table 3:** Relative abundance of (a) bacterial phyla (relative abundance > 1%) and (b) families (relative abundance > 2%*)* in soils under different treatments. Means (±standard deviations) in each phylum or family followed by similar letters are not significantly different at 5% probability level (Tukey’s HSD test).

(**a**)		**Broccoli**		**Faba bean**	
**Phylum**		*BR*_earth_	*BR*_no-earth_	*FB*_earth_	*FB*_no-earth_
Proteobacteria		29.41 ± 1.96 a	25.50 ± 1.14 bc	26.07 ± 1.47 b	22.71 ± 2.56 c
Bacteroidota		21.57 ± 0.84 a	18.66 ± 1.20 b	21.26 ± 0.92 a	18.74 ± 1.23 b
Actinobacteriota		12.23 ± 0.97 c	19.71 ± 1.55 a	15.58 ± 1.57 bc	17.58 ± 0.81 ab
Chloroflexi		8.50 ± 0.58 a	7.84 ± 0.60 a	6.42 ± 0.11 b	5.38 ± 0.19 b
Myxococcota		8.61 ± 0.46 a	5.16 ± 0.59 c	6.40 ± 0.25 b	4.85 ± 0.68 c
Planctomycetota		6.35 ± 0.58 a	6.32 ± 0. 63 a	6.33 ± 0.45 a	5.70 ± 0.41 a
Verrucomicrobia		3.60 ± 0.26 a	1.85 ± 0.16 c	2.57 ± 0.32 b	2.41 ± 0.32 bc
Firmicutes		3.35 ± 0.34 b	6.77 ± 0.75 a	4.56 ± 0.41 ab	6.78 ± 0.25 a
Patescibacteria		2.76 ± 0.19 c	3.17 ± 0.36 c	5.96 ± 0.24 b	9.68 ± 1.01 a
Acidobacteria		1.11 ± 0.22 d	1.37 ± 0.13 c	1.63 ± 0.06 b	2.03 ± 0.17 a
Gemmatimonadetes		0.95 ± 0.07 c	1.59 ± 0.22 ab	1.41 ± 0.25 b	1.70 ± 0.07 a
(**b**)		**Broccoli**		**Faba bean**	
**Phylum**	**Family**	*BR*_earth_	*BR*_no-earth_	*FB*_earth_	*FB*_no-earth_
Bacteroidota	Flavobacteriaceae	7.11 ± 0.76 b	7.97 ± 0.56 ab	10.24 ± 1.11 a	8.37 ± 0.59 ab
Myxococcota	BIrii41	6.19 ± 0.66 a	3.18 ± 0.24 c	4.69 ± 0.54 b	1.99 ± 0.39 c
Proteobacteria	Devosiaceae	5.34 ± 0.61 a	3.35 ± 0.19 b	2.76 ± 0.21 bc	2.20 ± 0.14 c
Planctomycetota	Pirellulaceae	4.43 ± 0.26 a	3.79 ± 0.45 b	4.12 ± 0.56 ab	3.41 ± 0.18 c
Bacteroidota	Microscillaceae	4.76 ± 0.35 a	3.36 ± 0.26 b	4.88 ± 0.24 a	4.12 ± 0.44 ab
Chloroflexi	SBR1031	2.77 ± 0.19 a	1.53 ± 0.22 b	1.13 ± 0.08 bc	0.64 ± 0.07 c
Actinobacteriota	Microbacteriaceae	2.44 ± 0.31 a	1.35 ± 0.21 b	1.34 ± 0.08 b	0.57 ± 0.08 c
Proteobacteria	R7C24	2.13 ± 0.18 a	0.72 ± 0.08 b	0.82 ± 0.09 b	0.33 ± 0.04 c
Firmicutes	Bacillaceae	1.29 ± 0.15 d	3.48 ± 0.23 b	2.15 ± 0.31 c	4.50 ± 0.39 a
Actinobacteriota	Streptomycetaceae	0.22 ± 0.04 c	1.84 ± 0.23 a	1.08 ± 0.17 b	2.04 ± 0.17 a
Bacteroidota	Saprospiraceae	0.30 ± 0.04 c	1.04 ± 0.21 b	0.70 ± 0.16 bc	2.38 ±\ 0.31 a
Actinobacteriota	Streptosporangiaceae	0.25 ± 0.04 c	2.04 ± 0.25 a	1.12 ± 0.21 b	2.41 ± 0.43 a

## Data Availability

The data presented in this study are available on request from the corresponding author.

## Supplementary Materials

The following supporting information can be downloaded at: https://www.mdpi.com/article/10.3390/plants12061216/s1. Table S1. Soil pH, soil organic carbon (SOC), soil total nitrogen (STN), litter organic carbon (LOC), litter total nitrogen (LTN), tea bag organic carbon (TOC) and tea bag total nitrogen (TTN) in green and red tea bags measured at the beginning of the trial (28 November). Each value represents the mean (± SD) from four measurements (*n* = 4). Table S2. Two-way ANOVA statistics of soil and litter parameters. Soil organic carbon (SOC); soil total nitrogen (STN); litter organic carbon (LOC); litter total nitrogen (LTN); organic carbon in green tea bags (TOCgreen); total nitrogen in green tea bags (TTNgreen); organic carbon in red tea bags (TOCred); total nitrogen in red tea bags (TTNred); soil carbon to nitrogen ratio (Soil C/N); litter carbon to nitrogen ratio (Litter C/N); carbon to nitrogen ratio in green tea bags (Green tea C/N); carbon to nitrogen ratio in red tea bags (Red tea C/N); soil pH; stabilization factor of tea bags (STBI); decomposition constant of tea bags (kTBI); percentage of litter decomposed (dlitter). Table S3. Effects of plant species (treatment A) and earthworms (treatment B), and their interaction effects (A × B) in a two-way ANOVA analysis on 16S rRNA gene copies number, bacterial phyla (relative abundance > 1%), and bacterial families (relative abundance > 2%). Values are represented as F values. * and **: significant at *p* < 0.05 and *p* < 0.01 level, respectively; ns: not significant. Figure S1. (row a) Earthworm weighting at the beginning of the trial and images shot on 1 December; (row b) images shot on 3 January; (row c) images shot on 1 February; (row d) images shot on 28 February; (row e) images shot on 15 March; (row f) images shot on 30 March. Red arrows indicate earthworms. Figure S2. (a) The four image scanners used and sealed; (b) two image scanners placed inside the pots; (c) the datalogger used for the measurement of soil temperature and soil water content; (d) board with integrated microcontroller; (e) temperature and capacitive sensors. Figure S3. Relative abundance (>1%) of bacterial families in soils under different treatments, consisting of broccoli with earthworms (BRearth), faba bean with earthworms (FBearth), broccoli without earthworms (BRno-earth), and faba bean without earthworms (FBno-earth). Ward's clustering method, using Euclidean distance, was used to estimate the distance among all bacterial families in response to the soil treatments. The measures within each treatment are in triplicate. Figure S4. Relative abundance (>1%) of bacterial genera in soils under different treatments, consisting of broccoli with earthworms (BRearth), faba bean with earthworms (FBearth), broccoli without earthworms (BRno-earth), and faba bean without earthworms (FBno-earth). Ward's clustering method, using Euclidean distance, was used to estimate the distance among all bacterial genera in response to the treatments. The measures within each treatment are in triplicate. Figure S5. ANCOM differential abundance volcano plot: BRearth (broccoli with earthworms) vs BRno-earth (broccoli without earthworms) The *x*-axis value represents the centered log ratio (clr) transformed F statistic (between groups). The *y*-axis value represents the empirical distribution of W (the number of times of the null-hypothesis was rejected. Taxa with rejected null-hypothesis are shown (p: phyla; c: class; o: order; f: family; g: genus). The measures within each treatment are in triplicate. Figure S6. ANCOM differential abundance volcano plot: FBearth (faba bean with earthworms) vs FBno-earth (faba bean without earthworms). The *x*-axis value represents the centered log ratio (clr) transformed F statistic (between groups). The *y*-axis value represents the empirical distribution of W (the number of times of the null-hypothesis was rejected. Taxa with rejected null-hypothesis are shown (p: phyla; c: class; o: order; f: family; g: genus). The measures within each treatment are in triplicate. Figure S7. 16S rRNA gene copies number in soils under different treatments, consisting of broccoli with earthworms (BRearth), broccoli without earthworms (BRno-earth), faba bean with earthworms (FBearth), and faba bean without earthworms (FBno-earth). Means with similar letters are not significantly different at 5% probability level (Tukey's HSD test). The measures within each treatment are in triplicate. Figure S8. Canonical Correspondence Analysis (CCA) showing the relationships between the bacterial community structure at phylum level (relative abundance > 1%) and the soil and litter parameters. The longer the lines identifying the parameters and the closer they are to the treatments, the more positively related to the treatments they are.
