# Supplementary material for: Earthworm-Driven Changes in Soil Chemico-Physical Properties, Soil Bacterial Microbiota, Tree/Tea Litter Decomposition, and Plant Growth in a Mesocosm Experiment with Two Plant Species
Source: Plants (Basel). 2023 Mar 7;12(6):1216. doi: 10.3390/plants12061216 (PMC10054492; doi:10.3390/plants12061216)
Supplement: Supplementary file 1 [file plants-12-01216-s001.zip › plants-2225226-supplementary.pdf]

Supplementary materials

Supplementary Table S1. Soil pH, soil organic carbon (*SOC*), soil total nitrogen (*STN*), litter organic carbon (*LOC*), litter total nitrogen (*LTN*), tea bag organic carbon (*TOC*) and tea bag total nitrogen (*TTN*) in green and red tea bags measured at the beginning of the trial (28 November). Each value represents the mean (± SD) from four measurements (n = 4).

**Soil pH**	***SOC*** **(g kg^{-1})**	***STN*** **(g kg^{-1})**	***LOC*** **(g kg^{-1})**	***LTN*** **(g kg^{-1})**	**TOC_{green} (g kg^{-1})**	**TTN_{green} (g kg^{-1})**	**TOC_{red} (g kg^{-1})**	**TTN_{red} (g kg^{-1})**
7.40 ± 0.03	43.3 ± 0.21	1.45 ± 0.02	40.8 ± 2.39	5.27 ± 0.87	39.7 ± 1.01	2.76 ± 0.09	43.0 ± 1.17	0.71 ± 0.02

Supplementary Table S2. Two-way ANOVA statistics of soil and litter parameters. Soil organic carbon (*SOC*); soil total nitrogen (*STN*); litter organic carbon (*LOC*); litter total nitrogen (*LTN*); organic carbon in green tea bags (TOC_{green}); total nitrogen in green tea bags (TTN_{green}); organic carbon in red tea bags (TOC_{red}); total nitrogen in red tea bags (TTN_{red}); soil carbon to nitrogen ratio (Soil C/N); litter carbon to nitrogen ratio (Litter C/N); carbon to nitrogen ratio in green tea bags (Green tea C/N); carbon to nitrogen ratio in red tea bags (Red tea C/N); soil pH; stabilization factor of tea bags (S_{TBI}); decomposition constant of tea bags (k_{TBI}); percentage of litter decomposed (d_{litter}).

Variable	*F* value - Earthworms	*P* value - Earthworms	*F* value - Plant species	*P* value - Plant species	*F* value - Earthworms × plant species	*P* value - Earthworms × plant species
SOC	33.543	0.00009	3.098	0.10381	7.428	0.01842
STN	18.450	0.00104	46.865	0.00002	5.838	0.03254
LOC	26.590	0.00024	11.662	0.00513	5.714	0.03411
LTN	385.457	0.00000	0.729	0.41004	0.102	0.75439
TOC_{green}	23.331	0.00041	0.002	0.96264	6.425	0.02619
TTN_{green}	19.497	0.00084	134.647	0.00000	6.074	0.02979
TOC_{red}	0.997	0.33785	14.857	0.00229	12.796	0.00380
TTN_{red}	0.369	0.55501	226.384	0.00000	0.223	0.64519
**Soil C/N**	0.096	0.76155	31.650	0.00011	8.889	0.01145
**Litter C/N**	121.392	0.00000	5.782	0.03325	2.642	0.13000
**Green tea C/N**	6.932	0.02186	12.047	0.00462	8.147	0.01451
**Red tea C/N**	2.498	0.14001	161.491	0.00000	10.896	0.00633
**Soil pH**	511.078	0.00000	1.916	0.19153	0.652	0.43515
S_{TBI}	7.871	0.01588	0.130	0.72486	0.292	0.59874
k_{TBI}	7.302	0.01923	0.313	0.58638	0.035	0.85527
d_{litter}	17.714	0.00121	0.009	0.92534	0.187	0.67331

Supplementary Table S3. Effects of plant species (treatment A) and earthworms (treatment B), and their interaction effects (A × B) in a two-way ANOVA analysis on 16S rRNA gene copies number, bacterial phyla (relative abundance > 1%), and bacterial families (relative abundance > 2%). Values are represented as *F* values. * and **: significant at $p < 0.05$ and $p < 0.01$ level, respectively; ns: not significant.

Variable	Plant species (A)	Earthworms (B)	A × B
16S rRNA gene copies number	0.29 ns	45.08 **	5.43 *
Bacterial phylum			
Proteobacteria	6.36 *	9.33 *	5.05 *
Bacteroidota	4.03 ns	18.00 **	5.09 *
Actinobacteriota	0.33 ns	19.64 **	6.57 *
Chloroflexi	47.58 **	6.64 *	5.33 *
Myxococcota	21.43 **	84.98 **	12.40 **
Planctomycetota	0.11 ns	0.12 ns	0.10 ns
Verrucomicrobia	1.34 ns	21.82 **	15.25 **
Firmicutes	3.89 ns	19.78 **	4.99 *
Patescibacteria	62.67 **	11.37 **	7.25 *
Acidobacteria	21.15 **	9.72 **	5.29 *
Gemmatimonadetes	11.10 *	30.03 **	5.19 *
Bacterial family			
Flavobacteriaceae	14.76 **	1.56 ns	10.10 *
BIrii41	17.04 **	76.58 **	5.23 *
Devosiaceae	53.62 **	24.95 **	7.78 *
Pirellulaceae	2.34 ns	4.33 ns	5.01 *
Microscillaceae	2.33 ns	13.93 **	4.23 ns
SBR1031	77.01 **	35.74 **	6.70 *
Microbacteriaceae	60.56 **	60.05 **	6.17 *
R7C24	137.69 **	174.10 **	40.73 **
Bacillaceae	4.66 ns	26.97 **	5.03 *
Streptomycetaceae	12.57 **	76.24 **	4.98 *
Saprospiraceae	39.88 **	76.34 **	11.53 **
Streptosporangiaceae	15.65 **	96.63 **	5.04 *

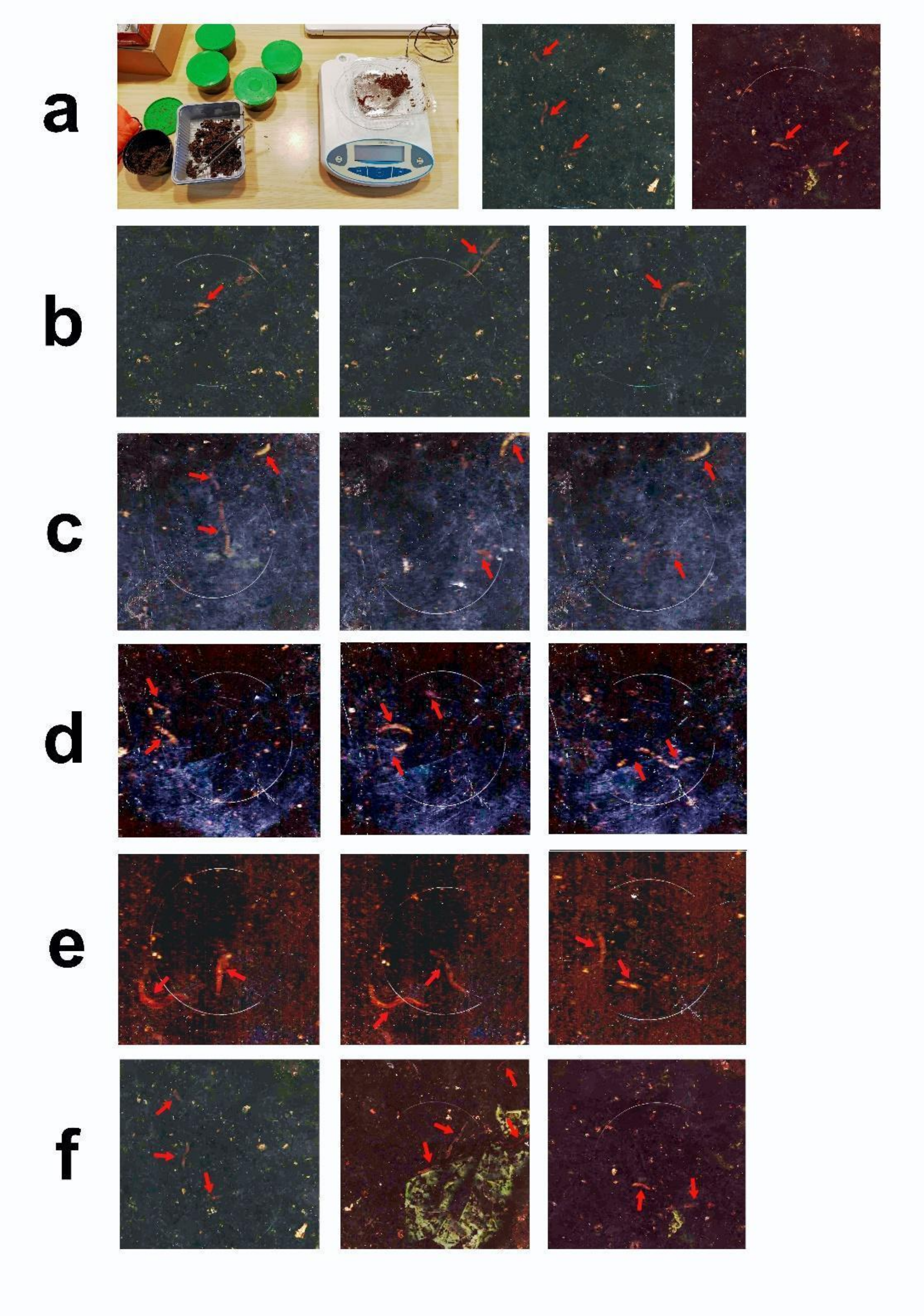

Supplementary Figure S1. (row a) Earthworm weighting at the beginning of the trial and images shot on 1 December; (row b) images shot on 3 January; (row c) images shot on 1 February; (row d) images shot on 28 February; (row e) images shot on 15 March; (row f) images shot on 30 March. Red arrows indicate earthworms.

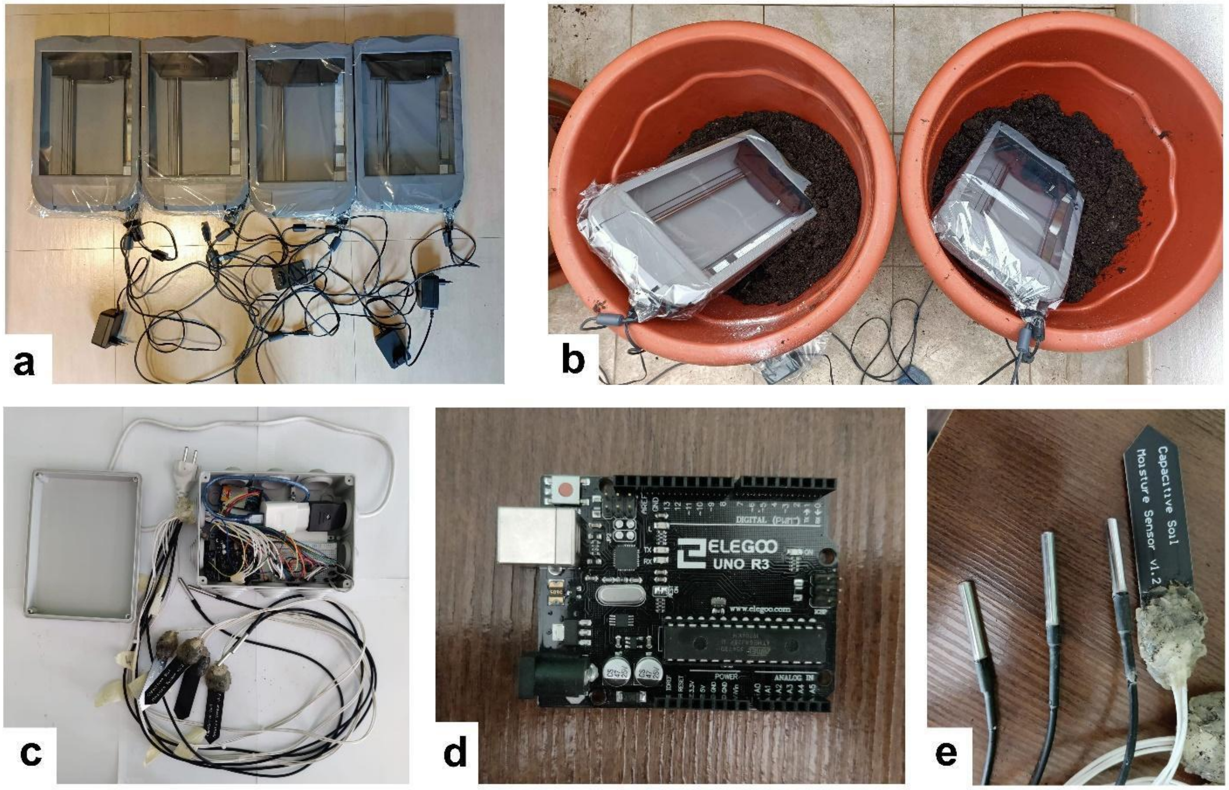

Supplementary Figure S2. (a) The four image scanners used and sealed; (b) two image scanners placed inside the pots; (c) the datalogger used for the measurement of soil temperature and soil water content; (d) board with integrated microcontroller; (e) temperature and capacitive sensors.

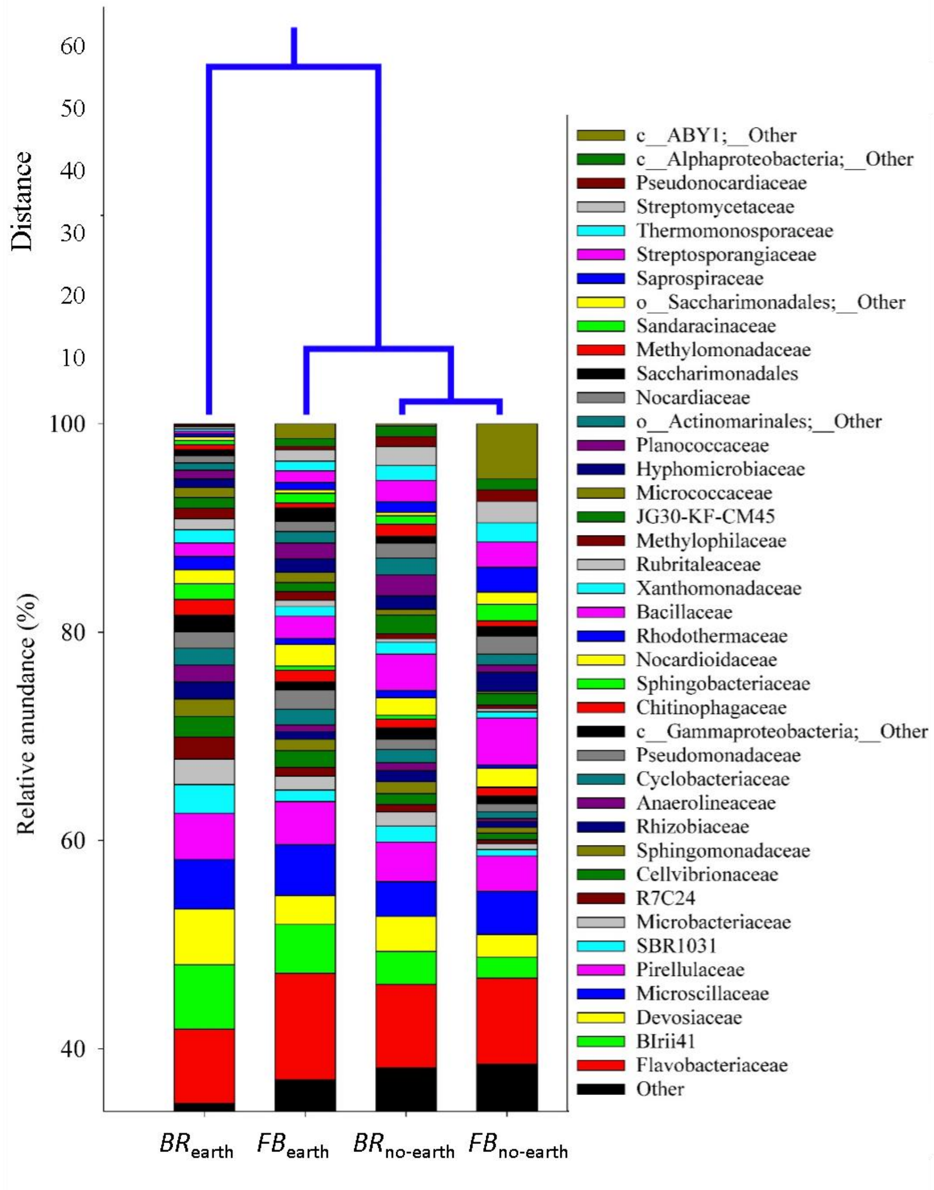

Supplementary Figure S3. Relative abundance (> 1%) of bacterial families in soils under different treatments, consisting of broccoli with earthworms (BR_{earth}), faba bean with earthworms (FB_{earth}), broccoli without earthworms ($BR_{\text{no-earth}}$), and faba bean without earthworms ($FB_{\text{no-earth}}$). Ward's clustering method, using Euclidean distance, was used to estimate the distance among all bacterial families in response to the soil treatments. The measures within each treatment are in triplicate.

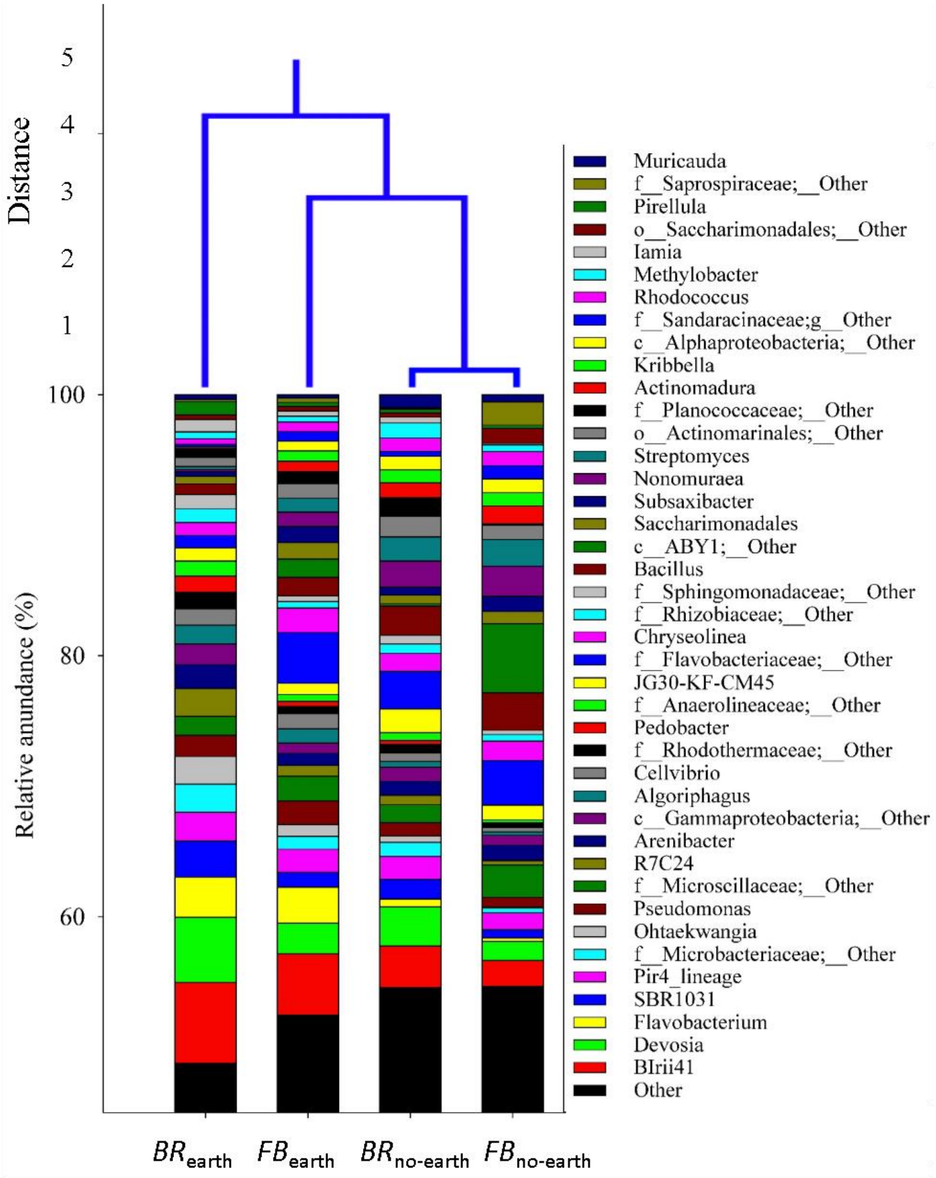

Supplementary Figure S4. Relative abundance (> 1%) of bacterial genera in soils under different treatments, consisting of broccoli with earthworms (BR_{earth}), faba bean with earthworms (FB_{earth}), broccoli without earthworms ($BR_{no-earth}$), and faba bean without earthworms ($FB_{no-earth}$). Ward's clustering method, using Euclidean distance, was used to estimate the distance among all bacterial genera in response to the treatments. The measures within each treatment are in triplicate.

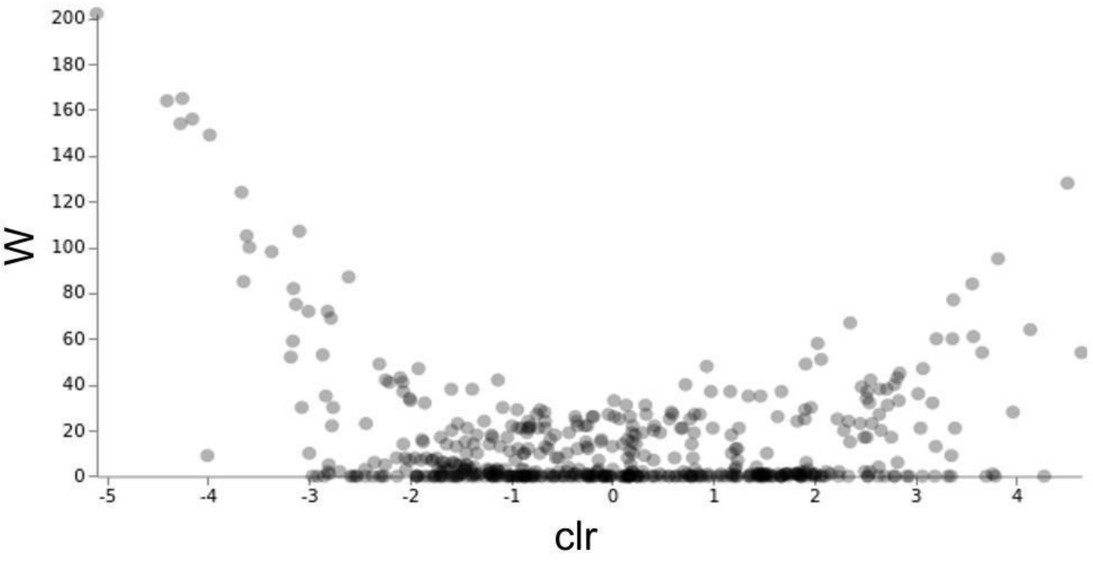

					W
p__Bacteroidota	c__Bacteroidia	o__Chitinophagales	f__Chitinophagaceae	g__Terrimonas	164
p__Proteobacteria	c__Gammaproteobacteria	o__Oceanospirillales	f__Pseudohongiellaceae	g__BIyi10	154
p__Verrucomicrobiota	c__Verrucomicrobiae	o__Opitutales	f__Opitutaceae	g__Opitutus	202
p__Verrucomicrobiota	c__Verrucomicrobiae	o__Verrucomicrobiales	f__Rubritaleaceae	g__Rubritalea	165
p__Verrucomicrobiota	c__Verrucomicrobiae	o__Verrucomicrobiales	f__Verrucomicrobiaceae	g__uncultured	156

Supplementary Figure S5. ANCOM differential abundance volcano plot: BR_{earth} (broccoli with earthworms) vs $BR_{no\text{-}earth}$ (broccoli without earthworms) The x-axis value represents the centered log ratio (clr) transformed F statistic (between groups). The y-axis value represents the empirical distribution of W (the number of times of the null-hypothesis was rejected. Taxa with rejected null-hypothesis are shown (p: phyla; c: class; o: order; f: family; g: genus). The measures within each treatment are in triplicate.

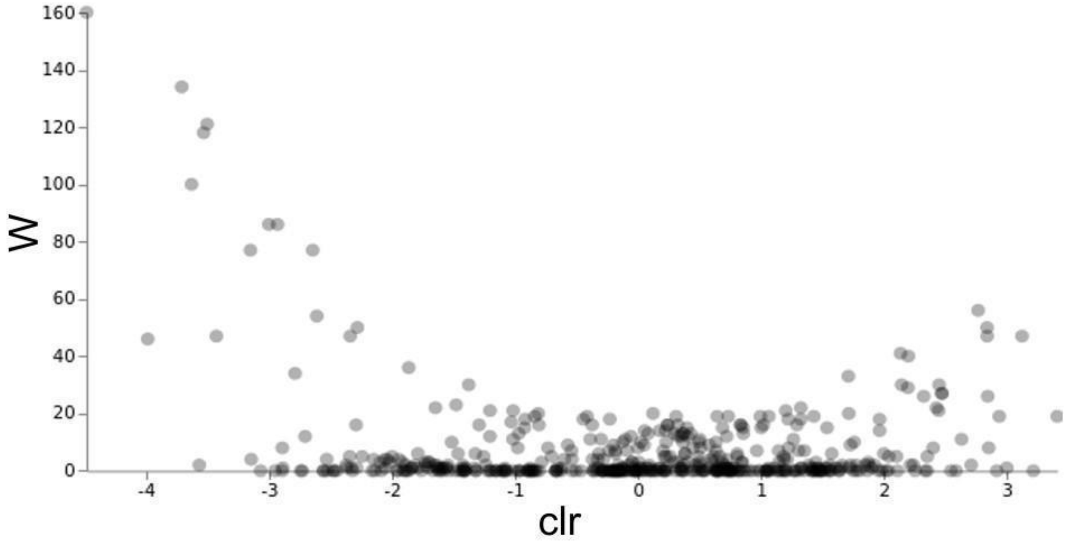

					W
p_Proteobacteria	c_Alphaproteobacteria	o_Rhodobacterales	f_Rhodobacteraceae	g_Paracoccus	160
p_Patescibacteria	c_Kazania	o_Kazania	f_Kazania	g_Kazania	134
p_Proteobacteria	c_Gammaproteobacteria	o_Burkholderiales	f_Comamonadaceae	g_Caenimonas	121
p_Planctomycetota	c_Phycisphaerae	o_S-70	f_S-70	g_S-70	118

Supplementary Figure S6. ANCOM differential abundance volcano plot: FB_{earth} (faba bean with earthworms) vs $FB_{no\text{-}earth}$ (faba bean without earthworms). The x-axis value represents the centered log ratio (clr) transformed F statistic (between groups). The y-axis value represents the empirical distribution of W (the number of times of the null-hypothesis was rejected. Taxa with rejected null-hypothesis are shown (p: phyla; c: class; o: order; f: family; g: genus). The measures within each treatment are in triplicate.

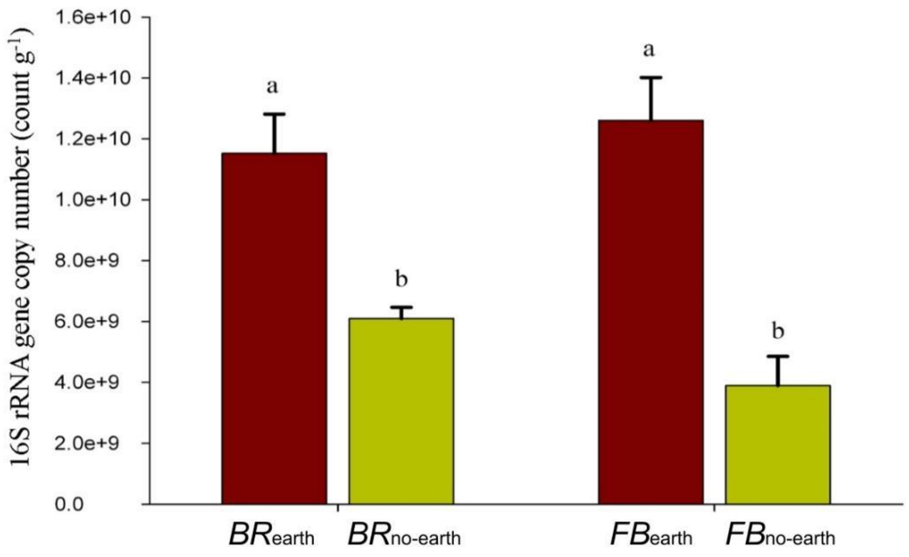

Supplementary Figure S7. 16S rRNA gene copies number in soils under different treatments, consisting of broccoli with earthworms (BR_{earth}), broccoli without earthworms ($BR_{no\text{-}earth}$), faba bean with earthworms (FB_{earth}), and faba bean without earthworms ($FB_{no\text{-}earth}$). Means with similar letters are not significantly different at 5% probability level (Tukey's HSD test). The measures within each treatment are in triplicate.

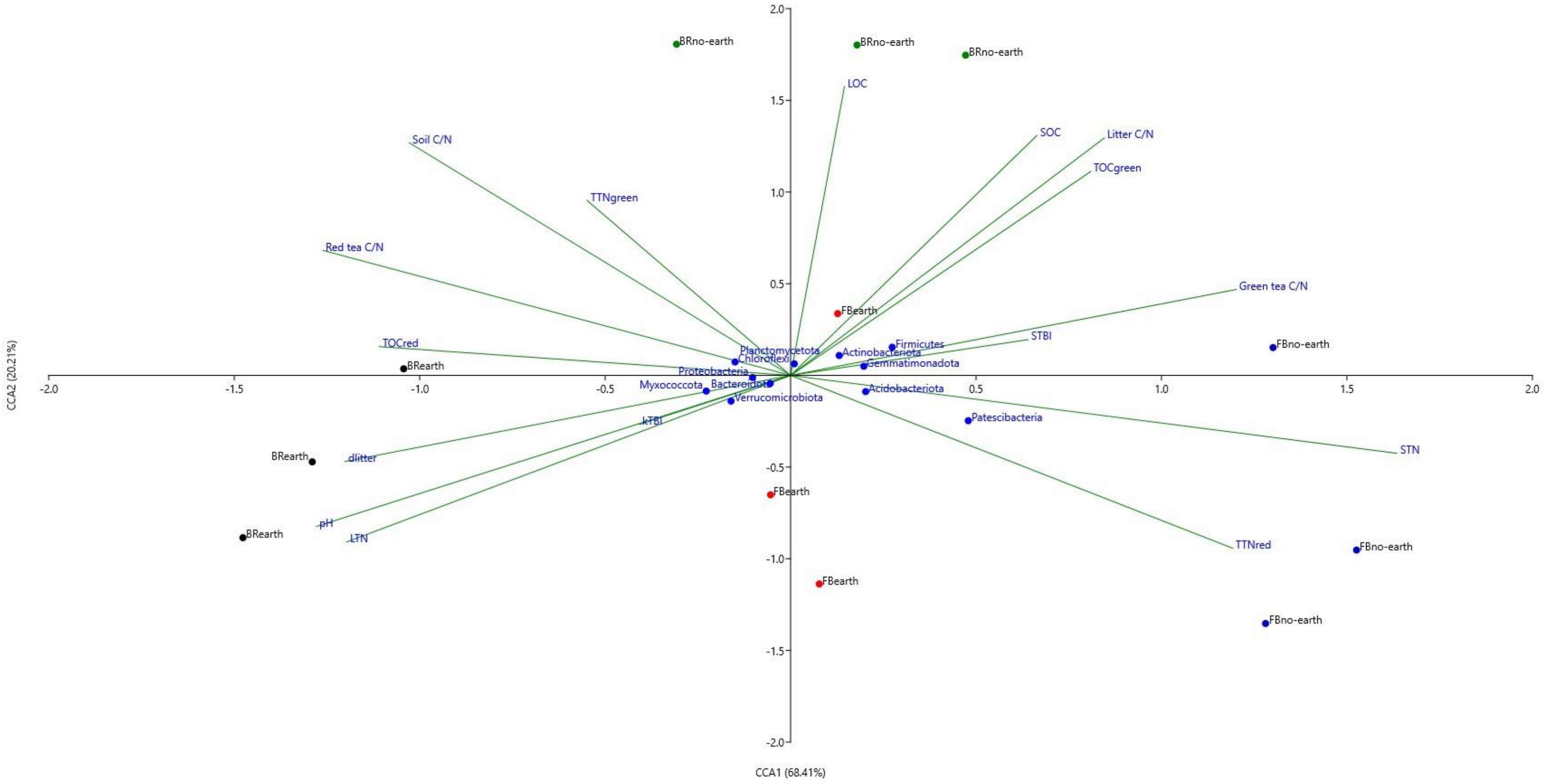

Supplementary Figure S8. Canonical Correspondence Analysis (CCA) showing the relationships between the bacterial community structure at phylum level (relative abundance > 1%) and the soil and litter parameters. The longer the lines identifying the parameters and the closer they are to the treatments, the more positively related to the treatments they are.
